# Anti-Parkinson Potential of Silymarin: Mechanistic Insight and Therapeutic Standing

**DOI:** 10.3389/fphar.2018.00422

**Published:** 2018-04-27

**Authors:** Hammad Ullah, Haroon Khan

**Affiliations:** Department of Pharmacy, Abdul Wali Khan University Mardan, Mardan, Pakistan

**Keywords:** silymarin, neuroprotective effects, anti-Parkinson’s activity, mechanistic insights, drug of future

## Abstract

Parkinson’s disease (PD) involves aggregation of α-synuclein and progressive loss of dopaminergic neurons. Pathogenesis of PD may also be related to one’s genetic background. PD is most common among geriatric population and approximately 1–2% of population suffers over age 65 years. Currently no successful therapies are in practice for the management of PD and available therapies tend to decrease the symptoms of PD only. Furthermore, these are associated with diverse range of adverse effects profile. The neuroprotective effects of polyphenols are widely studied and documented. Among phytochemicals, silymarin is one of the most widely used flavonoids because of its extensive therapeutic properties and has been indicated in pathological conditions of prostate, CNS, lungs, skin, liver, and pancreas. Silymarin is a mixture of flavonolignans (silybin, isosilybin, and silychristin), small amount of flavonoids (taxifolin), fatty acids, and other polyphenolic compounds extracted from the dried fruit of *Silybum marianum* and is clinically used for hepatoprotective effects since ancient times. Neuroprotective effects of silymarin have been studied in various models of neurological disorders such as Alzheimer’s disease, PD, and cerebral ischemia. The aim of the present study is to provide a comprehensive review of the recent literature exploring the effects of silymarin administration on the progression of PD. Reducing oxidative stress, inflammatory cytokines, altering cellular apoptosis machinery, and estrogen receptor machinery are mechanisms that are responsible for neuroprotection by silymarin, as discussed in this review. Additionally, because of poor aqueous solubility, the bioavailability of silymarin is low and only 23–47% of silymarin reaches systemic circulation after oral administration. Our primary focus is on the chemical basis of the pharmacology of silymarin in the treatment of PD and its mechanisms and possible therapeutic/clinical status while addressing the bioavailability limitation.

## Introduction

Parkinson’s disease is the most common neurodegenerative movement disorder characterized by progressive loss of dopaminergic neurons in substantia nigra pars compacta (SNpc) along with widespread intracellular aggregates of the protein α-synuclein (Thome et al., 2016; Pinto et al., 2018). Twenty genetic variants have been identified by human genetic studies, which are linked to PD pathogenesis (Pan et al., 2017). Currently monogenetic PD accounts for 3–5% of total cases of PD (Ben-David and Tu, 2015). PARK genes are most commonly linked to pathogenesis of PD and the inheritance patterns may be autosomal dominant such as in case of PARK 1, 3, 5, and 8 or autosomal recessive as in case of PARK 2, 6, and 7 (Hamza et al., 2010). The basic features of PD include tremors at rest, rigidity, bradykinesia, gait, and balance dysfunction (Patel et al., 2014). It has been observed that PD is found in all ethnic groups but geographical differences exist is prevalence of disease. Approximately 1–2% of the population suffers from PD over the age of 65 years and this figure increases to 3–5% in people of 85 years and older (De Lau and Breteler, 2006; Alves et al., 2008). The incidence rate of PD is 8–18 per 100,000 person-years. The rate of incidence is lower in Asian countries than in western countries (Tan, 2013; Lee and Gilbert, 2016). It is also documented that prevalence of PD will be almost double by 2030 and the burden of disease will also shift from developed western countries to developing eastern nations (Dorsey et al., 2007).

Although no successful therapies are currently available that can modify the disease. However, dopaminergic medications are the mainstay of treatment for symptomatic relief of motor symptoms (AlDakheel et al., 2014; Rizek et al., 2016). The available medications that are currently in practice for management of PD include levodopa, dopamine agonists (ropinirole, bromocriptine, cabergoline), MAOIs (selegiline), amantadine, anticholinergics (trihexyphenidyl), carbidopa, and entacapone (Nakaoka et al., 2014; Pagano et al., 2015). Levodopa is the most efficacious to control motor symptoms of the disease and is drug of choice to initiate first in course of treatment (Connolly and Lang, 2014). Other medications indicated for control of non-motor symptoms are clozapine and quetiapine for psychotic symptoms, SSRIs, TCAs, and SNRIs for depression, rivastigmine for dementia, BZs and non-BZ hypnotics for insomnia, and fiber-rich diet for constipation (Todorova et al., 2014). There are certain limitations to the use of anti-Parkinson’s medications especially to efficacious classes of drugs. Dopaminergic medications including levodopa are most commonly associated with psychosis, motor complications, and impulsive compulsive disorder (Rascol et al., 2003; Weiss and Marsh, 2012). Most of the patients with PD develop motor complications and dyskinesia within 5–10 years of levodopa treatment (Bãjenaru et al., 2016).

Polyphenols are most abundant antioxidant phytochemicals present in human diet. They are secondary metabolites present in foods and beverages of plant origin including fruits, vegetables, cereals, herbs, spices, legumes, nuts, olives, chocolate, tea, coffee, and red wine (Fantini et al., 2015). Polyphenols possess antimicrobial, anti-inflammatory, antiviral, anticancer, and immunomodulatory activities (Marzocchella et al., 2011; Benvenuto et al., 2013). Polyphenols could be divided in different classes depending on chemical skeleton of compound including phenolic acids, flavonoids, stilbenes, and lignans (Li et al., 2014; Kim et al., 2016). Polyphenols are capable of crossing blood–brain barrier and control neuronal disease pathogenesis at molecular and symptomatic level (Bhullar and Rupasinghe, 2013). The neuroprotective effects of polyphenols/natural compounds have been documented in various neurological disorders (Ayaz et al., 2015, 2017a,b; Ahmad et al., 2016; Patel et al., 2017; Rauf et al., 2017a,b; Farooq et al., 2018; Khan et al., 2018) including cerebral ischemia, brain edema, PD, amyotrophic lateral sclerosis, brain tumors, and cognitive impairments (Jagla and Pechanova, 2015). Their neuroprotective activities are attributed to their antioxidant potential, anti-inflammatory actions, and alteration of signaling pathways (Basli et al., 2012; Moosavi et al., 2016). While managing neurodegenerative, novel therapeutic strategies support the application on antioxidant polyphenols as monotherapy or antioxidant cocktail formulation (Vecchia et al., 2015).

Silymarin is the polyphenolic flavonoid extracted from dried fruit of *Silybum marianum* and is most commonly used for hepatoprotective activities since ancient times (Wang et al., 2015; AbouZid et al., 2016). Among phytochemicals it is one of the most widely used flavonoid because of its extensive therapeutic properties (Mady et al., 2016). Silymarin has been indicated in pathological conditions of various origins such as prostate, lungs, CNS, pancreas, and skin. It is considered safe at therapeutic doses but improper administration of dosages may lead to cause adverse drug reactions (ADRs) where gastrointestinal effects are more common among them (Karimi et al., 2011). Neuroprotective effects of silymarin have been studied in various models of neurological disorders such as Alzheimer’s disease, PD, and cerebral ischemia. Reducing oxidative stress, inflammatory cytokines, altering cellular apoptosis machinery, and estrogen receptor machinery are mechanisms that are responsible for neuroprotection by silymarin (Borah et al., 2013). Additionally because of poor aqueous solubility the bioavailability of silymarin is low and only 23–47% of silymarin reaches systemic circulation after oral administration (Yang et al., 2013). The aim of the present study is to provide comprehensive review of the recent literature exploring the effects of silymarin administration on progression of PD. Our primary focus is on the chemical basis of pharmacology of silymarin and its anti-Parkinson’s mechanisms.

## Chemistry

Silymarin is a mixture containing isomer flavonolignans (silybin, isosilybin, and silychristin), small number of flavonoids (taxifolin), fatty acids, and other polyphenolic compounds. It is a lipophilic agent extracted from seeds of *S. marianum.* Silybin comprises 50–70% of silymarin having greatest degree of biological activity (Ghosh et al., 2010; El-Elimat et al., 2014). Seeds of *S. marianum* also contain other flavonolignans including isosilybin, dehydrosilybin, desoxysilychristin, desoxysilydianin, silandrin, silybinome, silyhermin, and neosilyhermin (Karkanis et al., 2011). Flavonolignans present in the mixture of silymarin contain flavonoid moiety links to a molecule of lignin moiety (coniferyl alcohol) (Crocenzi and Roma, 2006). The molecular formula of flavonolignan skeleton is C_25_H_22_O_10_ and molecular weight is 482. Pelter and Hansel were first who established the structure of silybin in 1975 (Lee and Liu, 2003). It has been documented that silybin is a mixture of diastereoisomers namely silybin A and silybin B. Silybin also known as silibinin contains 1,4-dioxane ring in addition to flavonoid moiety and is a most active anti-hepatotoxic agent. It has been reported that the presence of 2,3-double bond in the C-ring of flavonoid structure results in increasing antioxidant activity of silybin (Ahmed et al., 2003; Ramasamy and Agarwal, 2008; Khan et al., 2011).

## Sources

**FIGURE 1:**
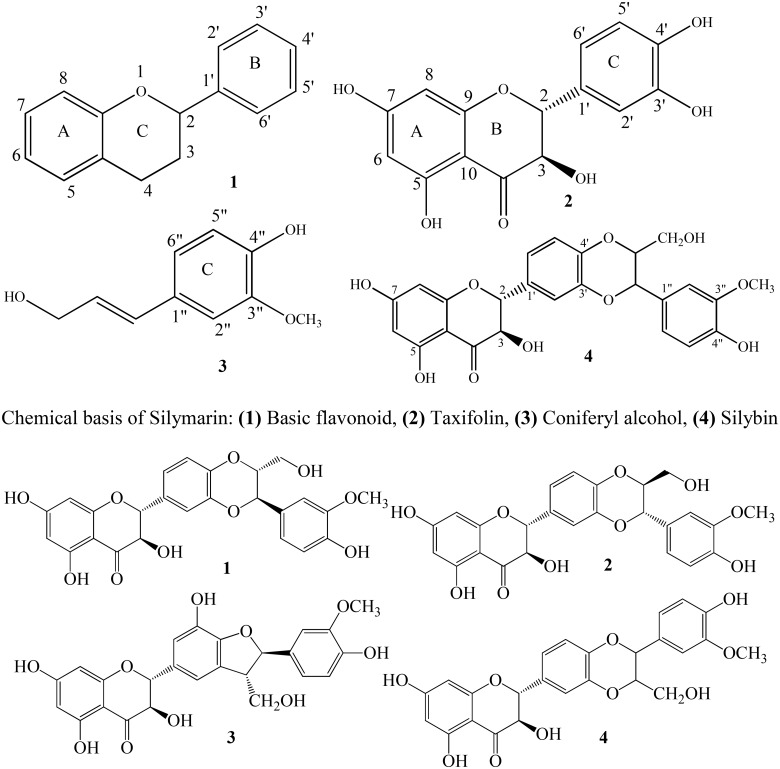
Structures of silymarin compounds: 1, silybin A; 2, silybin B; 3, silychristin; and 4, isosilybin.

Silymarin (**Figure 1**) is a pharmacologically active phytochemical extracted from seeds and fruits of *S. marianum*, commonly known as milk thistle (Wagoner et al., 2010; Wang et al., 2015). *S. marianum* is an annual or a biennial plant and is a member of plant family Asteraceae (**Table 1**). The genus *Silybum* contains two species that are *S. marianum* and *Silybum eburneum*. Geographically this plant distributes around the globe. It is cultivated in Mediterranean region, Sinai, Afghanistan, and has been neutralized in other parts of the world (Karkanis et al., 2011; AbouZid et al., 2016). It has been used from ancient times where Theophrastus (4th century B.C.) was probably first to describe it under the name Pternix. The initial use of *S. marianum* was reported by Dioscorides for treatment of serpent bites. In 1898, the use of herb to relieve obstructions of the liver was documented by British herbalist Culpepper (Post-White et al., 2007; Karkanis et al., 2011). Today, it is one of the most commonly used botanical supplement in the world. As reported in 2012, it ranked sixth in total US botanical supplement sales (Kawaguchi-Suzuki et al., 2014).

**Table 1:** Components of silymarin mixture.

Botanical source	Local name	Constituents	Quantity (%)	Reference
*Silybum marianum*	Milk thistle	Silybin A	16	(Davis-Searles et al., 2005; Ross, 2008; Polyak et al., 2013)
		Silybin B	24	
		Isosilybin A	6	
		Isosilybin B	4	
		Silydianin	16	
		Silychristin	12	
		Isosilychristin	2	
		Taxifolin	2	

## Pathophysiology of Parkinson’S Disease

**FIGURE 2:**
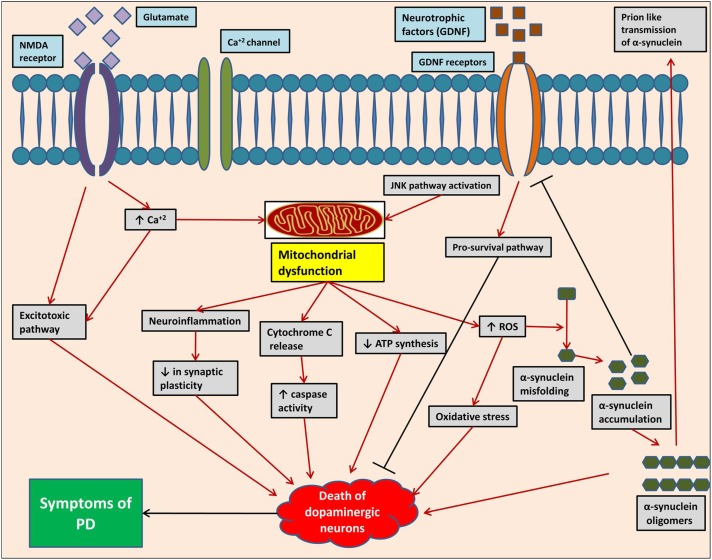
Pathogenesis of Parkinson’s disease at molecular level, showing different complex mechanistic singling pathway/events.

The neuropathological mechanisms (**Figure 2**) responsible for pathogenesis of PD include protein misfolding, disrupted protein handling, mitochondrial dysfunction, oxidative stress, impaired calcium handling, and neuroinflammation (Brundin and Melki, 2017). The substantia nigra (SN) and ventral tegmental area (VTA) are major dopaminergic nuclei located in mid-brain playing most important functions of brain. The progressive loss of dopaminergic neurons in SN pars compacta and dopaminergic denervation in forebrain areas is the main characteristic of motor symptoms of PD (Politis, 2014; Jovanovic et al., 2018). There is also non-dopaminergic neuronal loss contributing to non-motor symptoms of PD. These neurons include monoaminergic cells in the locus coeruleus and raphe nuclei, cholinergic cells in the nucleus basalis of Meynert, pedunculopontine tegmental nucleus, and hypocretin cells in the hypothalamus. Loss of cholinergic cells, pedunculopontine tegmental nucleus, and hypocretin cells are associated with cognitive dysfunction, gait problems, and sleep disorders, respectively, as seen in PD. It has been reported that the presence of 140 amino acid containing proteinaceous α-synuclein-rich inclusions called as Lewy bodies exclusively in neurons is closely related with neuronal loss in PD (Leverenz et al., 2009; Jowaed et al., 2010; Obeso et al., 2010; Surmeier et al., 2017).

Several factors either from genetic background or environmental factors play a crucial role in cellular α-synuclein misfolding. Mitochondrial dysfunction, oxidative stress, failure of liposomal autophagy, ubiquitin proteasome system, and neuroinflammation are factors responsible for α-synuclein misfolding and cell-to-cell transfer of pathogenic α-synuclein assemblies (Brundin and Melki, 2017). α-Synuclein pathology is a neurotoxic process in which the formation of oligomers occurs from soluble α-synuclein monomers, which then combines to form large insoluble α-synuclein fibrils (Kim and Lee, 2008; Melki, 2015). Liposomal autophagy and ubiquitin proteasome are both responsible for α-synuclein degradation and thus impairment of these degradation systems can contribute to α-synuclein accumulation (Xilouri et al., 2013; Kaushik and Cuervo, 2015). Like other neurodegenerative disorders, formation of reactive oxygen species (ROS) is a key mechanism in pathogenesis of PD causing degeneration of dopaminergic neurons. Metabolism of dopamine, mitochondrial dysfunction neuroinflammation, high level of iron and calcium in SN pars compacta, and aging all are contributing factors in the generation of ROS. At mitochondrial level, the electron transport chain is the major contributor in causing oxidative stress. Other sources of ROS formation are monoamine oxidase (MAO), NADPH oxidase, and other flavo-enzymes along with nitric oxide (NO). Lipid peroxidation also occurs under oxidative stress as brain contains high concentration of polyunsaturated fatty acids. Mutations in α-synuclein, parkin, PINK1, DJ-1, and LRRK2 are related to mitochondria and thus studies linked these mutations with oxidative stress (Dias et al., 2013; Yan et al., 2013).

Neuroinflammation may not be an initial trigger, but it is one of the most essential factor in pathogenesis of PD. As documented, catecholaminergic and dopaminergic neurons express MHC-I proteins which can expose them to cytotoxic T-cell-mediated death in the presence of antigens. Activation of complement system on Lewy bodies pre-exposes neurons to inflammatory processes (Loeffler et al., 2006; Poewe et al., 2017). Glial cells activation in SN pars compacta has been reported concurrently with an increased expression of pro-inflammatory mediators. CCAAT/enhancer binding protein ß (C/EBPß) is a transcription factor whose role in pathogenesis of PD has been reported. It regulates the expression of genes involved in inflammatory processes and brain injury (Morales-Garcia et al., 2017). Studies suggest important role of cyclooxygenase-2 (COX-2) enzyme in secondary activation of microglia, in the progression of the inflammatory response, and in the progressive loss of dopaminergic neurons (Vijitruth et al., 2006).

## Neuroprotective Potential of Silymarin

Silymarin is a polyphenolic flavonoid with strong antioxidant activities and is in clinical practice for management of hepatic disorders. Free radicals scavenging, elevating cellular glutathione level, and improving activity of superoxide dismutase are key mechanisms attributed to antioxidant activities of silymarin. Through inhibition of oxidative stress, silymarin possesses neuroprotective effects and it can be used in the management of neurodegenerative disorders including Alzheimer’s disease, PD stroke, and traumatic brain injury (Kittur et al., 2002; Chtourou et al., 2010; Muley et al., 2012; Pandima Devi et al., 2017). It has been reported that silymarin inhibits the activation of microglia as well as production of inflammatory mediators such as tumor necroses factor-alpha (TNF-α) and NO, as a result reducing damage to dopaminergic neurons (Křen and Walterova, 2005; Kaur et al., 2011). As reported, silymarin maintained striatal dopamine levels by diminishing apoptosis in the SN and preserving dopaminergic neurons. Studies linked these effects with antioxidant and anti-inflammatory potential of silymarin (Abushouk et al., 2017). It has been also documented from some studies that silymarin reduces level of α-synuclein protein and increasing dopamine level (Srivastava et al., 2017).

## Molecular Mechanisms of Silymarin

**FIGURE 3:**
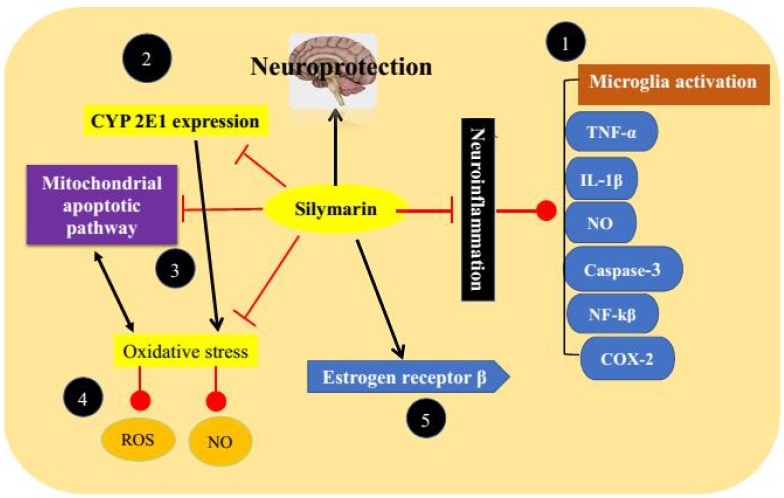
Anti-Parkinson’s targets of silymarin. (1) By acting through inhibition of neuroinflammation, (2) inhibition of CYP 2E1 expression leads to free radical formation, (3) inhibition of mitochondrial apoptotic pathway, (4) prevention of oxidative stress, and (5) modulation of estrogen receptors.

Different researchers have extensively investigated the molecular mechanisms of silymarin in various experimental models of neurological and PD (**Figure 3**).

### Antioxidant Potential

Silymarin has already been reported to protect neurons against oxidative stress and nitrosative stress by elevating both enzymatic and non-enzymatic antioxidant markers. It is capable of inhibiting the formation of oxygen and peroxyl radicals along with the protein oxidation products (Galhardi et al., 2009; Borah et al., 2013). Silybin in the mixture of silymarin is mainly responsible for its antioxidant activities. However, it has been documented that a mixture of silymarin components showed higher antioxidant activities because of synergistic effects (Nabavi et al., 2012; Pérez-H et al., 2014; Pérez-Hernández et al., 2016). Silymarin treatment can reduce levels of LDH, NO, and ROS as well as oxidants/antioxidants balance (Chtourou et al., 2011). It results in elevation of glutathione levels by increasing transcriptional and translational activities of proteins used to synthesize glutathione, decreased degradation of glutathione, increased reduction of glutathione disulfide, and by increasing transport of precursors (Johnson et al., 2012). Silymarin also down regulates the expression of CYP 2E1 which induces free radical generation by mixed function oxidase activity and thus reduces oxidative stress (Singhal et al., 2011). Furthermore, it maintains mitochondrial integrity and function and inhibits mitochondrial apoptotic pathway (Fernández-Moriano et al., 2015).

### Anti-inflammatory Activities

As stated earlier in this review that neuroinflammation is a consequence or a cause of nigral cell loss and thus plays one of the most crucial roles in pathophysiology of PD. Beside antioxidant properties silymarin also inhibits neuroinflammation by several mechanisms (Tansey and Goldberg, 2010; Machado et al., 2011). Silymarin exerts its anti-inflammatory activities mainly by inhibiting microglia activation. It reduces the production of inflammatory mediators such as TNF-α, IL-1β, and NO, and protects dopaminergic neurons against degeneration (Wang et al., 2002; Stojkovska et al., 2015). It has been documented that silymarin inhibits the production of inducible NO synthase in a dose-dependent manner. No production in neuroglial cells surrounding neurons has been correlated with neurotoxicity and pathogenesis of neurodegenerative disorders. Silymarin inhibits NO production at <300 ppm (Gazak et al., 2007; Matés et al., 2009). It decreases striatal levels of caspase-3, NF-kβ, and down regulates COX-2 reflecting its anti-apoptotic and anti-inflammatory activities (Haddadi et al., 2014; Olson and Gendelman, 2016). Activated caspase-3 is responsible for apoptosis of dopaminergic neurons and thus its reduction can protects against apoptosis (Yamada et al., 2010). It inhibits the activation of NF-κB by decreasing phosphorylation of p65 subunit which is responsible for strong transcription activating potential of NF-κB (Gu et al., 2007; Lee et al., 2014).

### Anti-Parkinson’s Potential: *In Vitro* Studies

Neuroprotective actions of silymarin have been reported using several *in vitro* models of PD. *In vitro* studies reported that antioxidant and anti-inflammatory activities of silymarin are basically responsible for its neuroprotection (Matkowski, 2008; Ramasamy and Agarwal, 2008). Lipopolysaccharide (LPS) is most widely used as neurotoxic agent *in vitro* models of PD. It protects dopaminergic neurons from LPS-induced neurotoxicity by inhibiting activation of microglia reflecting its anti-inflammatory actions (Kittur et al., 2002; Hald and Lotharius, 2005; Lee et al., 2014). It has been documented from *in vitro* studies that silymarin also reduces superoxide and TNF-α production while inhibiting inducible NO synthase (Wang et al., 2002). NF-κB pathway plays an important role in pathogenesis of inflammation by regulating pro-inflammatory cytokine production, leukocyte recruitment, or cell survival (Lawrence, 2009). Silymarin regulates NF-κB 100 times better than aspirin. Several kinases regulate NF-κB that belong to mitogen-activated protein kinase (MAPK) family and C-Jun N-terminal kinase (JNK). Silymarin inhibits these kinases without posing any threat to the cell (Kidd, 2009; Lawrence, 2009).

### Anti-Parkinson’s Potential: *In Vivo* Studies

The potential of silymarin in management on PD has been reported from several *in vivo* studies. *In vivo* models of PD show that activation of caspases in microglia leads to initiation of inflammatory cascade results in degeneration of dopaminergic neurons (Antonietta Panaro and Cianciulli, 2012). LPS exposure results in overproduction and over expression of cytokines and chemokines including IL-8, IL-1β, TNF-α, and playing a significant role in the pathogenesis of PD (Tentillier et al., 2016). *In vivo* models also suggest that MPTP treatment results in over expression of pro-inflammatory cytokine receptors (Lofrumento et al., 2011). Silymarin by diminishing apoptosis in the SN preserve dopaminergic neurons and thus maintain striatal dopaminergic levels (Pérez-H et al., 2014). It has been reported that silymarin normalizes gene expression of up-regulated NF-κB1 and caspase-9 (Raza et al., 2011; Singhal et al., 2013). However, studies also suggest that silymarin induces many features of apoptosis in *Candida albicans* such as disruption of calcium homeostasis, loss of MMP, DNA fragmentation, and caspase activation. It needs further research whether these effects have a link with neurological actions of silymarin (Lee and Lee, 2018). The binding affinity of silymarin for estrogen receptor β in CNS regions has also been reported. Estrogen attenuates toxin-induced neurotoxicity, prevents lipid peroxidation, acting synergistically with antioxidants such as glutathione (Singh et al., 2006; Baluchnejadmojarad et al., 2010).

### Safety Profile of Silymarin

Being a phytochemical silymarin generally possesses favorable safety profile, although allergic reactions including anaphylactic reactions have been reported (Geier et al., 1990). Other ADRs include mild laxative effects, nausea, epigastric discomfort, arthralgia, pruritus, urticaria, and headache (Mayer et al., 2005). Silymarin also leads to inhibition of cytochrome P450 system and thus affecting the clearance of other drugs including chemotherapeutic agents (Venkataramanan et al., 2000). However, no interaction found with cisplatin, doxorubicin, vincristine, and L-asparaginase in pre-clinical studies at concentrations used. These effects may be dose related require further study at higher doses (Post-White et al., 2007).

### Low Solubility of Silymarin

Low solubility of silymarin has been documented, i.e., 0.04 mg/ml and this is one of the basic reason of low oral bioavailability of silymarin from GIT. Studies also reflect that, however, silymarin has low aqueous solubility, it possess no lipophilic properties (Woo et al., 2007). Several strategies have been investigated that can improve the solubility and bioavailability of Silymarin including self-microemulsifying drug delivery systems (SMEDDS), solid dispersions, porous silica nanoparticles (PSNs), and liposomes (Yang et al., 2015). SMEDDS results in 3.6 times increase in bioavailability comparatively to reference capsule (Woo et al., 2007). A novel solid dispersion system containing silymarin, polyvinylpyrrolidone (PVP), and Tween-80 have been increased drug solubility by about 650-folds with physical and chemical stability of 6 months (Hwang et al., 2014). The PSNs are a novel approach to improve the bioavailability of drugs with poor solubility such as silymarin. The prepared PSNs consist of narrow pore size distribution of approximately 10 nm. Silymarin-loaded PSNs showed an initial burst release followed by sustained release over a period of 72 h (Cao et al., 2012). Incorporating silymarin in liposomal carrier system gave increase in AUC and Cmax and thus showed better hepatoprotective and anti-inflammatory effects when compared to silymarin suspension (Kumar et al., 2014).

## Concluding Remarks

Silymarin is a polyphenolic phytochemical with promising therapeutic potential. It is extracted from dried fruit of *S. marianum* and has been used as a hepatoprotective agent since long time. Later-on, the beneficial role of silymarin in the treatment of pathological conditions of various origins including cancer has been studied and documented. It is a mixture of flavonolignans with strong antioxidant and anti-inflammatory activities. It also has binding affinity with estrogen receptor β in CNS regions which attenuates neurotoxicity and prevents lipid peroxidation. These effects make silymarin a valuable choice in therapeutics of neurodegenerative disorders such as PD. Additionally, it has shown significant neuroprotective effects in various *in vitro* and *in vivo* models. However, because of low aqueous solubility the bioavailability of silymarin is quite low i.e., 23–47%. Several techniques are available to improve the bioavailability of silymarin such as SMEDDS, solid dispersions, PSNs, and liposomes. Moreover, the bioavailability issue can be resolved via chemical derivatization. Thus, further research is required on these grounds in order to get molecule of clinical utility for the treatment of PD.

## Author Contributions

All authors listed have made a substantial, direct and intellectual contribution to the work, and approved it for publication.

## Conflict of Interest Statement

The authors declare that the research was conducted in the absence of any commercial or financial relationships that could be construed as a potential conflict of interest.
